# Preliminary in vitro antiplasmodial activity of *Aristolochia griffithii* and *Thalictrum foliolosum* DC extracts against malaria parasite *Plasmodium falciparum*

**DOI:** 10.1186/s13104-016-1862-4

**Published:** 2016-01-28

**Authors:** N. G. Das, Bipul Rabha, P. K. Talukdar, Diganta Goswami, Sunil Dhiman

**Affiliations:** Defence Research Laboratory, Tezpur, Assam 784001 India

**Keywords:** *Aristolochia griffithii*, *Thalictrum foliolosum* DC, Plant extracts, Antimalarial, *Plasmodium*

## Abstract

**Background:**

Resistance development in human malaria parasites against commonly used antimalarial drugs has necessitated the scientific exploration of traditionally used antimalarial plants. Plant derivatives have been used for curing malaria historically. The present study involves in vitro evaluation of two medicinally important plants *Aristolochia griffithii* and *Thalictrum foliolosum* DC used in antimalarial chemotherapy by the tribes of northeast India.

**Method:**

Chloroform, ethyl acetate and n–butanol extracts of *Aristolochia griffithii* and *Thalictrum foliolosum* DC were evaluated in vitro against chloroquine sensitive (SS) and chloroquine resistance strains (RS) of *P. falciparum*. The tests were conducted following WHO standard method and the inhibition of parasite (IC_50_) was calculated.

**Results:**

In *A. griffithii*, the IC_50_ value for ethyl acetate extracts against SS was 6.2 ± 0.02 μg/ml and found to be lower than chloroform extracts, which exhibited an IC_50_ value of 14.1 ± 0.1 μg/ml (t = 191.1; p < 0.0001). The IC_50_ values of both chloroform and ethyl acetate extracts for RS were higher as compared to the SS (p < 0.0001). In *T. foliolosum* DC the IC_50_ concentration of chloroform extracts for SS and RS were 0.5 ± 0.0 and 1.1 ± 0.0 μg/ml respectively (t = 54.2; p < 0.0001).

**Conclusion:**

The present findings, although preliminary, but scientifically demonstrate that identification and isolation of active compounds of these two plant materials and testing against different *Plasmodium* species could lead to the development of potential antimalarial drugs for future.

## Background

Approximately 214 million cases and 0.44 million deaths were reported due to malaria worldwide in 2015 [1]. *Plasmodium falciparum*, the life threatening malaria parasite, has developed resistance to many antimalarials, which has entailed the urgent need of developing new and effective antimalarial drugs that are affordable to the developing countries [2–7]. Medicinal plants have been a source of many antimalarial compounds such as quinine, artemisinin that have been used in developing potential antimalarial drugs. Artemisinin derivatives have been recommended in combination with other antimalarial drugs, such as amodiaquine, mefloquine, sulphadoxine–pyrimethamine (SP) for the treatment of malaria in most of the endemic countries. In order to identify potential antimalarial compounds, several studies have been conducted in malaria endemic countries to evaluate the suppressive effects of various plant derivatives on malaria parasite [8–10].

Malaria is commonly reported in many states of India and neighbouring countries and the problem of reduced susceptibility to commonly used antimalarial drugs specifically in *P. falciparum* has been a growing concern for malaria control programme in India [11, 12]. In addition to the threat to life, malaria has been found to have detrimental effect on the prosperity of the society by impeding the overall development, including effect on population growth, productivity and medical cost [13]. In India, the resistance to chloroquine was first detected in Assam, since then it prevailed into other parts of the country attributing countless malaria related casualties. The tremendous pressure of chloroquine resistance has led to switch-over to artesunate-based combination therapy (ACT) as first line of treatment of uncomplicated malaria cases. Therefore, it is important that traditionally used antimalarial plants are investigated to discover new antimalarial drugs, in order to tackle the resurging of antimalarial resistance in *Plasmodium* parasite. In the recent years, various studies have explored the antimalarial activity of phyto-drugs based on the traditional reputation of plants in malaria treatment [2, 8–10, 14, 15].

Northeast region of India has great plant biodiversity potential and many plants are used in traditional medicine system for the treatment of various ailments [16–19]. Among the tribes of Assam, especially Bodo, Karbi, Mishing and Dimasa, the plant *Aristolochia griffithii* is used against insect and snake bite, skin problem, stomach problems and fever. However, Adi and Monpa tribes of Arunachal Pradesh use *Thalictrumfoliolosum* DC for the treatment of nematode worms, stomach problem, fever and pain. Both the plants materials are used traditionally in treatment of malaria by the tribals of the region but experimental data to support the activity against malaria parasite is lacking. Therefore after collecting basic information about antimalarial activity of both the plant materials from the local tribals, we have evaluated the anti-plasmodial activity of *A. griffithii,* Hook and *T. foliolosum* DC extracts in vitro against *P. falciparum* in the present study.

## Methods

### Plants and chemicals

*Aristolochia griffithii* Hook, commonly called Nagbal, was collected from Kalamati foot Hills of Sonitpur district of Assam, whereas *Thalictrum foliolosum* DC, commonly called Yangchira, was collected from the hills of Bomdila, West Kameng district of Arunachal Pradesh (Table 1). The aqueous extracts of root portion of both the plants has been used in the traditional treat of malaria for many years. Therefore, roots were used as testing material in the present study. The information about the use of plants in malaria treatment was gathered from locals through group discussions. The plants used in the study were identified by Dr. Ashish Kar of Tata Energy Research Institute, Guwahati, India and the voucher specimens of the plant material have been preserved in Defence Research Laboratory, Tezpur, India. Chloroform, ethyl acetate and n-butanol extracts were prepared using HPLC grade chemicals obtained from Merck, India Ltd., whereas water extracts were prepared using Millipore (Merck Millipore, USA) filtered water.

**Table 1:** Details of plants used in anti-malarial evaluation against *P. falciparum*

Name	Local name	Part used	Collection time	Location (district)	GPS coordinates
*Aristolochia griffithii*, Hook	Nagbal	Root	Nov–Dec 2009	Kalamati foot hills (Sonitpur)	N 26°51′06″ E 92°30′12″
*Thalictrum foliolosum*, DC	Yangchira	Root	Oct–Dec 2009	Bomdila (West Kameng)	N 27°19′11″ E 92°26′05″

### Preparation of extracts and maintenance of *P. falciparum* culture

Plant materials were dried under the shade and grinded using an electric grinder. One hundred gram of individual material was macerated into 1 L of each solvent and thoroughly mixed using a sterilized glass rod at room temperature. The solution was kept for 24 h and mixed after every 2 h. Extracts were filtered through Whatman No. 1 filter paper, freeze dried and kept at 4 °C in well-closed containers for use in the anti-plasmodial assay. The extracts were dissolved in dimethylsulfoxide (DMSO) at concentration of 1 mg/ml and then diluted with incomplete medium (without serum) to achieve required concentrations of 0.1, 0.5, 1, 5, 10, 20 and 30 μg/ml for evaluation against known *P. falciparum* chloroquine sensitive strain, 3D7 (SS) and chloroquine resistance strains, LS1 (RS). *P. falciparum* culture was maintained in vitro on human erythrocytes (blood group O^+^) in RPMI-1640 medium (Sigma) supplemented with 10 % AB^+^ human serum, 25 mM N-2-hydroxyethylpiperazine-N′-2-ethanesulfonic acid (HEPES buffer; Sigma Aldrich), 25 mM NaHCO_3_ and 60 µg/ml gentamicin sulfate (7.2 pH) at 37 °C and 5 % CO_2_ in a CO_2_ incubator (HERA cell 240i, Thermo Scientific).

### *P. falciparum* sensitivity study

*Plasmodium falciparum* was synchronized for the ring stages [20] and diluted with fresh uninfected human RBC’s to adjust the level of parasitaemia to 1000–8000/µl of blood to study the sensitivity against plant extracts. The tests were done in 96 well microtitre plates for 24–36 h in duplicate following standard methods [21, 22]. An aliquot of parasite culture (100 µl) was added into the each well of the plate and then 100 µl of incomplete culture medium containing extracts at various concentrations was added into the well plates. Titre plates were kept in CO_2_ incubator at 37 °C for 24–36 h. After 24–36 h, the growth was monitored by preparing thick smear from each test as well as from the control well. The films were stained 10 % Giemsa solution (pH 7.3) and observed under light microscope (100×). The number of schizonts was counted per 200 asexual stages of parasite and values were compared between test wells and control well. The percent inhibition of parasite was calculated as follows:

$${\text{Inhibition}}=100-{\text{S}}$$

where S = (No. of schizonts in the test well/No. of schizonts in control well) × 100 [23].

The concentration at which 50 % inhibition obtained was recorded as IC_50_ value and determined using log dose probit method.

### Data analysis

Comparison of IC_50_ values among sensitive and resistant strain and two extracts was done using paired students ‘t’ test, while among different extracts were made using one way Krusal Wallis (K) test following Dunn test of multiple comparison. All the data obtained were tested for normality using Kolmogorov and Smirnov (KS) method.

## Results

The results of in vitro antimalarial evaluation of *A. griffithii* have been shown in (Table 2). In SS, the IC_50_ concentration for ethyl acetate extracts was 6.2 ± 0.02 μg/ml and found to be statistically lower than the chloroform extracts, which exhibited IC_50_ value of 14.1 ± 0.1 μg/ml (t = 191.1; p < 0.0001). Similarly, for RS, the IC_50_ value for ethyl acetate extracts was lower as compared to chloroform extracts, however the difference was not very much significant (t = 3.6; p = 0.005). Furthermore, the IC_50_ values of both chloroform and ethyl acetate extracts for RS were higher as compared to the SS (t = 49.6; p < 0.0001 for chloroform; t = 223.8; p < 0.0001 for ethyl acetate). Water soluble extracts and n-butanol extracts of *A. griffithii* did not show considerable activity. The IC_50_ values of water extracts for SS and RS strains were 274.0 ± 8.2 and 390.3 ± 1.7 respectively, while the IC_50_ concentration of n-butanol extracts for SS and RS were 90.7 ± 1.1 and 139.7 ± 0.3 μg/ml respectively.

**Table 2:** In vitro IC_50_ values of *A. griffithii* extracts against *P. falciparum*

Extract	SS (95 % CI)	RS (95 % CI)	t (p)
Chloroform	14.1 ± 0.1 (14.0–14.2)	16.2 ± 0.0 (16.2–16.3)	46.6 (<0.0001)
Ethyl acetate	6.2 ± 0.0 (6.2–6.2)	16.0 ± 0.1 (15.9–16.2)	223.8 (<0.0001)
n-Butanol	90.7 ± 1.1 (89.9–91.4)	139.7 ± 0.3 (138.0–141.3)	77.2 (<0.0001)
Water	274 ± 8.2 (248.1–299.9)	390.3 ± 1.7 (384.8–395.7)	13.9 (<0.0001)

SS, *P. falciparum* sensitive strain; RS, *P. falciparum* resistant strain; IC_50_ values in μg/ml; 95 % CI, 95 % confidence interval

The extracts of *T. foliolosum* DC were also found to be effective against *P. falciparum* in the present study (Table 3). The IC_50_ concentration of *T. foliolosum* DC chloroform extracts for SS and RS were 0.5 ± 0.0 and 1.1 ± 0.0 μg/ml respectively, which differed statistically (t = 54.2; p < 0.0001). For SS, the IC_50_ value was found to be lowest in chloroform extracts and higher in ethyl acetate extracts (K = 21.7; p < 0.0001). The chloroform extracts and n-butanol extracts showed antimalarial inhibition at very low concentration, however significant different was observed between the IC_50_ values of both the extracts for SS (t = 189.3; p < 0.0001) and RS (t = 405.1; p < 0.0001). The water soluble extracts exhibited an IC_50_ concentration of 11.0 ± 0.1 and 10.8 ± 0.1 μg/ml for SS and RS respectively, indicating that the activity of water extracts was higher against RS as compared to the SS (t = 5.8; p = 0.001).

**Table 3:** In vitro IC_50_ values of *T. foliolosum* DC extracts against *P. falciparum*

Extract	SS (95 % CI)	RS (95 % CI)	t (p)
Chloroform	0.5 ± 0.0 (0.5–0.5)	1.1 ± 0.0 (1.0–1.1)	54.2 (<0.0001)
Ethyl acetate	14.9 ± 0.1 (14.8–15.0)	26.0 ± 0.2 (25.9–26.2)	151.4 (<0.0001)
n-Butanol	3.4 ± 0.0 (3.4–3.5)	7.3 ± 0.0 (7.3–7.4)	190.9 (<0.0001)
Water	11.0 ± 0.1 (10.9–11.1)	10.8 ± 0.1 (10.6–10.9)	5.8 (0.001)

SS, *P. falciparum* sensitive strain; RS, *P. falciparum* resistant strain; IC_50_ values in μg/ml; 95 % CI, 95 % confidence interval

## Discussion

Although many antimalarial drugs have been proved to be very effective against malaria parasite, but emerging resistance to these drugs have highlighted the need for new therapeutic agents, which could replace existing antimalarial drugs, if required. In this investigation, in vitro anti-plasmodial activity of two Indian medicinal plants, *A. griffithii* and *T. foliolosum* DC root extracts was studied against a well known human malaria parasite. The plant material extracts were found to be effective against SS as well as RS of *P. falciparum* parasite. In *A. griffithii,* the chloroform and ethyl acetate extracts showed IC_50_ values of 14.1 ± 0.1 and 6.2 ± 0.0 for SS and 16.2 ± 0.0 and 16.0 ± 0.10 for RS respectively. However the IC_50_ values for n-butanol and water extracts were significantly high as compared to the chloroform and ethyl acetate extracts, indicating that both chloroform and ethyl acetate extracts have high parasiticidal activity and were more effective against the malaria parasite. *A. griffithii* is a shrub climbing plant with heart shape leaves, approximately 10–28 cm long, 8–26 cm wide and distributed at an altitude of 2000–2900 m. This plant is perennial, deciduous and fragrant and has abundant distribution in Arunachal Pradesh and Sikkim states of northeastern India. Many species of *Aristolochia* has been used in traditional medicine for the treatment of seizures, snakebite, intestinal pain, gallbladder pain, arthritis, gout, rheumatism, eczema, weight loss and wounds, however the antimalarial activity of this plant has not been evidenced by any scientific experimentation [24].

Further, in *T. foliolosum* DC, the chloroform and n-butanol extracts were more effective than ethyl acetate and water soluble extracts against both SS and RS of *P. falciparum*. The chloroform extracts of *T. foliolosum* DC were found to have highest activity against *P. falciparum* in present study, as the IC_50_ values for chloroform extracts against SS and RS were 0.5 ± 0.0 and 1.1 ± 0.0 respectively. *T. foliolosum* DC is a perennial plant with a height of about 2.5 m. The plant is distributed in the temperate Himalayas from 1500 to 2400 m, in the Khasi hills and in Kashmir, Punjab, Delhi, Uttar Pradesh, Bihar and Orissa. The root contains alkaloids, which has a stimulant action on the movements of the gastrointestinal tract, a depression of both the auricles and ventricles, distinct dilatation of the heart and induces hypotension [17]. *T. foliolosum* DC is a medicinally important plant and has been used as antipyretic, diuretic, febrifuge, ophthalmic, purgative and stomachic. It is considered to be a good remedy for atonic dyspepsia and is also useful in treating peptic ulcers, indigestion, toothache, hemorrhoids, for convalescence after acute diseases and liver disorders. The juice of the leaves is applied to boils and pimples [17]. Many studies have evaluated the antimalarial activity of plant derivatives and indicated their efficacy against the *Plasmodium* species using in vivo and in vitro methods [2, 8–10, 14, 15, 18, 19]. A recent study conducted in Thailand has suggested the promising activity of *Plumbago indica* Linn against both chloroquine resistant and chloroquine-sensitive strains of *P. falciparum* [25]. The study also indicated that the activity of plumbagin was relatively higher against chloroquine-resistant *P. falciparum* as compared to the chloroquine-sensitive *P. falciparum,* which suggests that the plants based compound may be more useful in clearing the resistant malaria parasite.

The majority of extracts used in the current study displayed convincing in vitro anti-plasmodial activity against known chloroquine susceptibility and resistant strains. The aqueous extracts and decoction of both the plant materials are traditionally used in malaria treatment in the study areas, however the organic extracts displayed excellent anti-plasmodial activity currently compared to aqueous counterparts. The present study using two indigenous plants has indicated that both have intrinsic anti-plasmodial activity. These plants have been used in the treatment of human malaria for many years by the tribes of northeastern India, however the current results have provided the scientific reason behind the folkloric use of *A. griffithii* and *T. foliolosum* DC in the treatment of malaria.

## Conclusion

Continuous spreading of multi-drug resistant malaria parasite has entailed that there is a need of exploring and evaluating new antimalarial molecules to combat malaria. Current study, aimed for searching new and effective anti-plasmodial drugs, indicated the potential in vitro antimalarial activity of *A. griffithii* and *T. foliolosum* DC collected from the northeastern region of India. Although these plants have been used in traditional medicine system of the region, but the antimalarial activity has been demonstrated for the first time. The selected extracts of the plants were very effective against SS and RS of *P. falciparum* in laboratory condition, which may be exploited for the treatment of chloroquine sensitive as well as resistance malaria patients. The present findings, however preliminary, but scientifically evidences the antimalarial potential of both the plants evaluated currently. Identification, isolation of active compound and testing against different *Plasmodium* species could lead to the development of more effective antimalarials for the future.

## Abbreviations

SS: chloroquine sensitive strain
RS: chloroquine resistance strain
IC: inhibitory concentration
WHO: World Health Organisation
μg: microgram
ml: mililitre
SP: sulphadoxine–pyrimethamine
ACT: artesunate-based combination therapy
HPLC: high performance liquid chromatography
DMSO: dimethylsulfoxide
RPMI: Roswell Park Memorial Institute medium
RBC: red blood corpuscles
KS: Kolmogorov Smirnov
